# Measurement invariance analysis of the Postpartum Specific Anxiety Scale - Research Short Form in mothers of premature and term infants

**DOI:** 10.1186/s13104-024-06746-3

**Published:** 2024-03-14

**Authors:** Semra Worrall, Paul Christiansen, Asma Khalil, Sergio A. Silverio, Victoria Fallon

**Affiliations:** 1 Department of Psychology, Institute of Population Health, University of Liverpool, Liverpool, UK; 2 Fetal Medicine Unit, Liverpool Women’s NHS Foundation Trust, Liverpool, UK; 3 Fetal Medicine Unit, St. George’s University Hospitals NHS Foundation Trust, University of London, London, UK; 4 Department of Women & Children’s Health, School of Life Course & Population Sciences, King’s College London, London, UK; 5 School of Psychology, Faculty of Health, Liverpool John Moores University, Liverpool, UK

**Keywords:** Gestational age, Preterm birth, Postpartum anxiety, Psychometric measurement, Measurement invariance

## Abstract

**Objective:**

Mothers of premature infants are more likely to develop anxiety during the first postpartum year than mothers of term infants. However, commonly used measures of anxiety were developed for general adult populations and may produce spurious, over-inflated scores when used in a postpartum context. Although perinatal-specific tools such as the Postpartum Specific Anxiety Scale [PSAS] offer a promising alternative form of measurement, it is not clear whether the measure performs similarly in mothers of premature infants as it does in mothers of term infants. The objective of the current study was to identify whether items on the Postpartum Specific Anxiety Scale - Research Short Form (PSAS-RSF) are being interpreted in the same manner in mothers of term infants and mothers of premature infants. Mothers (N = 320) participated in an international on-line survey between February 2022 and March 2023 (*n* = 160 mothers of premature infants, *n* = 160 mothers of term infants) where they completed the PSAS-RSF. Data were analysed using a measurement invariance analysis to assess whether constructs of the PSAS-RSF are performing in a similar manner across the two groups.

**Results:**

Whilst the PSAS-RSF achieved configural invariance and so retains its four-factor structure, metric invariance was not reached and so items are being interpreted differently in mothers of premature infants. Items concerning infant-separation, finance, and anxieties surrounding infant health are potentially problematic. Future research must now modify the PSAS-RSF for specific use in mothers of premature infants, to ensure measurement of anxiety in this population is valid.

## Introduction

Whilst many women report the transition to motherhood positively, for some, it can present psychological, social, physical, and emotional challenges. The transition to motherhood has been suggested as a potential period of psychological vulnerability [1], with anxiety and depression often highlighted as examples of poor mental health in the postnatal period.

Preterm birth represents a significant public health issue. The World Health Organization [WHO] estimate that over 13 million infants were born too early in 2020, with preterm birth now the leading cause of child mortality worldwide [2]. Adverse consequences include respiratory diseases [3], and increased likelihood of neuro-developmental delay comparative to term infants [4]. 

Research consistently shows mothers who give birth prematurely are more likely to develop depression [5], post-traumatic stress disorder [6], and anxiety [7], when compared to mothers of term infants. Postpartum anxiety in particular, may be exacerbated in mothers of premature infants compared to mothers of term infants, perhaps due to the sudden and unexpected nature of the birth [8], and uncertainty surrounding infant health [9]. 

Postpartum anxiety can be characterised by excessive, irrational worries occurring during the postpartum. From an evolutionary perspective, some anxieties after childbirth are adaptive, so there is a need to evaluate the presence of anxieties occurring after birth that may be considered maladaptive [10], through effective screening and identification. Despite high prevalence rates (up to 40%) [11], postpartum anxiety remains under-recognised and under-diagnosed [12]. This may perhaps be, in part, due to the lack of appropriate measurement tools for postpartum women. Most studies measuring postpartum anxiety use tools validated in general adult populations, which have been extrapolated for use in mothers as there has historically been no suitable alternative [13]. This can lead to spurious findings which may be unreliable [14]. 

Recently, the Postpartum Specific Anxiety Scale [PSAS] has been developed for research purposes and validated as a 51-item long-form [15], and as 16-item (PSAS-RSF) [16], and 12-item (PSAS-RSF-C) [17] short-forms. The PSAS has not been widely used in mothers of premature infants; to date only one study has investigated preterm birth and anxiety using the measure [7]. It is hypothesised however, some items contained within the PSAS may not be interpreted in the same manner in mothers of premature infants as with mothers of term infants. Items particularly pertinent to mothers of preterm infants may produce inflated findings given the increased likelihood of health concerns in this population [18], irrespective of anxiety. Similarly, items assessing anxieties surrounding infant development and maternal self-efficacy may also be elevated, given these are common anxieties for mothers of premature infants [19, 20]. 

Given the vast social, economic, and psychological consequences of preterm birth, it is essential to investigate if using a postpartum-specific tool performs in a similar manner in mothers of premature infants as it does in mothers of term infants to ensure accurate measurement. This study therefore aims to undertake a measurement invariance analysis [21], to assess if anxiety has the same meaning across groups, using the PSAS-RSF in an English-speaking population of mothers of preterm and term infants in the first postpartum year.

## Materials and methods

The current study forms part of a wider survey investigating postpartum anxiety and gestational age. The present analysis comprises datasets from two studies, which received full ethical approval from the University of Liverpool Institute of Population Health Research Ethics Committee (Study 1 ref:- 10606; Study 2 ref:- 3616). Written informed consent was gained via an electronically signed consent form, and participants received a full debrief upon completion.

Participants were recruited on-line, via social media and by word-of-mouth. Participants who were under 18 years of age, whose infant was born < 24 week’s gestation, or who were currently in the Neonatal Intensive Care Unit [NICU], were not eligible to participate. Participants were eligible to take part in the study if they were over the age of 18, had an infant born > 24 week’s gestation. If participants had an infant in the NICU, they had to have been discharged and well for at least three months to take part. Study 1 data were collected at two separate time points: February to August 2022 and November 2022 to March 2023. Study 1 data were combined with Study 2 data, which had been collected between January and February 2023, rendering 320 participants total. This allowed for the creation of two groups of equal number (premature [*n* = 160]; and term [*n* = 160]). There is no specific power calculation for measurement invariance, but the sample size was determined following the rule of 10 cases per item [22], rendering 160 participants for each group as there are 16 items on the PSAS-RSF. Participants completed demographic questions and psychometric scales including the PSAS-RSF [16]. 

## Measures and analysis plan

### Demographics

Several demographic questions, including age, ethnicity, and educational attainment were asked. Infant demographics, including gestational age (week of birth), and birth order were also gathered.

### Postpartum Specific Anxiety Scale - Research Short Form (PSAS-RSF)

The PSAS-RSF [16] is a 16-item scale developed in 2021 measuring the frequency of maternal-and-infant anxieties occurring during the previous seven days, scored on a Likert scale from 1 (not at all) to 4 (almost always). The scale can be used in mothers of infants from 0 to 12 months of age. It features four subscales, consisting of four items each, scored separately: Maternal Competence and Attachment Anxieties, Practical Infant Care Anxieties, Infant Safety and Welfare Anxieties, and Psychosocial Adjustment to Motherhood. The scale has excellent reliability in the current study (McDonald’s ω_*h*_ = 0.75).

### Method of analysis

All data analyses were conducted in R version 4.2.1, using lavaan [23], dplyr [24], corrplot [25], semTools [26], semTables [27], and psych [28] packages. Measurement invariance of the scale was tested across the two groups (mothers of term and mothers of premature infants). Diagonally weighted least squares estimation was used as individual responses on the PSAS-RSF are ordinal, scored on a scale of one-to-four [29].

Configural invariance was tested to investigate if the factor structure held across the two groups, with a random effect of group in the model. Model fit was assessed using several indices. The Tucker-Lewis Index [TLI] and Comparative Fit Index [CFI] were computed, with values of ≥ 0.95 considered excellent [30]. A Root Mean Square Error of Approximation [RMSEA] was also computed, with values of < 0.05 considered good, and values of 0.08 considered fair [30, 31]. Finally, the Standardised Root Mean Square Residual [SRMR] was also computed, with values of < 0.08 considered good [30].

The configural model was then compared to a metric invariance model. The model structure remained the same, but factor loadings across groups are fixed, which allows interpretation as to whether each individual item on the scale performs in the same way across the two groups. The validity of this model was tested using CFI differences (ΔCFI < 0.01), RMSEA differences (ΔRMSEA < 0.015), and SRMR differences (ΔSRMR < 0.03) as cut-offs [32]. If problematic, that is: values are above these figures, loadings across items were allowed to vary, to attempt to reach metric invariance. Problematic items were identified by assessing differences in standardised regression coefficients between the two groups.

The metric model is then compared to the scalar invariance model whereby factor loadings and intercepts are assumed to be equal across the two groups, so means of factor loadings can be compared. The cut-offs remain the same as the metric invariance model, but the SRMR cut-off is stricter (ΔSRMR < 0.015).

## Results

### Participants

Mothers in both the premature (*n* = 160) and term (*n* = 160) groups were predominantly from the United Kingdom of Great Britain and Northern Ireland and the Republic of Ireland (52% vs. 59%), married (63% vs. 81%), primiparous (66% vs. 76%); with both groups demonstrating similar levels of education to either undergraduate or equivalent degree level (34% vs. 39%). See Tables 1 and 2 for full demographic characteristics.

**Table 1:** Maternal demographic characteristics, split by the premature and term groups

Maternal Characteristic	Value		Maternal Characteristic	Value	
	Premature (*N* = 160)	Term (*N* = 160)		Premature (*N* = 160)	Term (*N* = 160)
*Maternal Age (M ± SD)*	30.76 ± 5.74	29.31 ± 4.22	*Current Diagnosis of Clinical Anxiety (N/%)*		
			Yes	84 (52.50%)	59 (36.90%)
*Country of Residence (N/%)*			No	72 (45.00%)	101 (63.10%)
			Prefer Not to Say	4 (2.50%)	0 (0.00%)
United Kingdom of and Great Britain and Northern Ireland and Ireland	84 (52.50%)	95 (59.40%)			
United States	49 (30.60%)	58 (36.30%)	*Timing of Anxiety Diagnosis* ^*b*^ *(N/%)*		
Other European	4 (2.50%)	0 (0.00%)	Before Pregnancy	44 (27.50%)	46 (28.70%)
Other Non-European	23 (14.38%)	7 (4.38%)	During Pregnancy	7 (4.40%)	4 (2.50%)
			Postpartum	33 (20.60%)	9 (5.60%)
*Ethnic Origin (N/%)*					
White	141 (88.10%)	147 (91.90%)	*Taking Prescribed Medication for Anxiety* ^*b*^ *(N/%)*		
Pakistani	2 (1.30%)	0 (0.00%)	Yes	44 (27.50%)	7 (4.40%)
Black Caribbean and Black African	8 (5.00%)	1 (0.60%)	No	39 (24.40%)	52 (32.50%)
Indian	3 (1.90%)	1 (0.60%)	Declined to Answer	1(1.19%)	0 (0.00%)
Other	6 (3.80%)	10 (6.25%)			
Prefer Not to Say	0 (0.00%)	1 (0.60%)	*Current Diagnosis of Clinical Depression (N/%)*		
			Yes	60 (37.50%)	14 (8.80%)
*Marital Status (N/%)*			No	99 (61.90%)	146 (91.30%)
Married	101 (63.10%)	129 (80.60%)	Prefer not to Say	1 (0.60%)	0 (0.00%)
Living with Partner	44 (27.50%)	27 (16.90%)			
Separated	3 (1.90%)	0 (0.00%)	*Timing of Depression Diagnosis* ^*b*^		
Single	12 (7.50%)	4 (2.50%)	Before Pregnancy	29 (18.10%)	11 (6.90%)
			During Pregnancy	5 (3.10%)	3 (1.90%)
*Occupation (N/%)*			Postpartum	26 (16.30%)	14 (8.80%)
Managers, Directors and Senior Officials	11 (6.90%)	5 (3.10%)			
Professional Occupations	52 (32.50%)	53 (33.10%)	*Taking Prescribed Medication for Depression* ^*b*^		
Associative Professional and Technical Occupations	2 (1.30%)	9 (5.60%)	Yes	38 (23.80%)	7 (4.40%)
Administrative and Secretarial Occupations	18 (11.30%)	20 (12.50%)	No	21 (13.10%)	7 (4.40%)
Skilled Trades Occupations	3 (1.90%)	9 (5.60%)	Declined to Answer	1(1.67%)	0 (0.00%)
Caring, Leisure and Other Service Occupations	16 (10.00%)	51 (31.90%)			
Sales and Customer Service Occupations ^a^	14 (8.8%)	N/A	*Maternal Anxiety on PSAS-RSF (M ± SD)*		
Process, Plant and Machine Operatives	0 (0.00%)	4 (2.50%)	Maternal Competence and Attachment Anxieties	7.85 ± 2.80	6.95 ± 2.06
Elementary Occupations	11 (6.90%)	1 (0.60%)	Infant Safety and Welfare Anxieties	11.49 ± 3.11	8.98 ± 2.52
Not in a Paid Occupation	33 (20.60%)	8 (5.00%)	Practical Infant Care Anxieties	10.78 ± 2.86	8.24 ± 2.46
			Psychosocial Adjustment to Motherhood	11.52 ± 2.82	9.25 ± 2.36
*Educational Attainment (N/%)*			PSAS-RSF Total	41.64 ± 9.04	33.41 ± 6.46
Completed Postgraduate Education, or equivalent	38 (23.80%)	19 (11.90%)			
Completed Undergraduate Education, or equivalent	55 (34.40%)	63 (39.40%)			
Completed A-Levels, or equivalent	29 (18.10%)	47 (29.40%)			
Completed GCSE’s, or equivalent	17 (10.60%)	28 (17.50%)			
No Qualifications	7 (4.40%)	1 (0.60%)			
Other	14 (8.80%)	2 (1.30%)			
*Housing Situation (N/%)*					
Own your Own Home	89 (55.6%)	120 (75.00%)			
Rent Privately	50 (31.30%)	28 (17.50%)			
Rent from the Local Authority	13 (8.10%)	7 (4.40%			
Live with Parents	3 (1.90%)	3 (1.90%)			
Other	5 (3.10%)	2 (1.30%)			
*Occupant Number, inc. Participant (N/%)*					
Two	10 (6.30%)	5 (3.1%)			
Three	86 (53.80%)	115 (71.90%)			
Four	38 (23.80%)	22 (13.8%)			
Five or more	26 (16.25%)	18 (11.30%)			

***N/B.***

^a^ This option was not presented to participants in the term group.

^b^ This item was only presented to participants who answered ‘Yes’ to the current clinical diagnosis question. Participants were free not to answer the questions.

^c^ Participants (*n* = 15) from the second dataset were not presented with these questions. N/percentages are therefore representative of the *n* = 145 participants who were presented these questions.

**Table 2:** Infant demographic characteristics, split by the premature and term groups

Infant Characteristic	Value	
	Premature (*N* = 160)	Term (*N* = 160)
*Infant Age (M ± SD)*	21.54 ± 14.86	20.37 ± 12.92
*Multiple Birth (N/%)*		
Yes	18 (11.30%)	3 (1.90%)
No	142 (88.80%)	157 (98.1%)
*Birth Order*		
First	106 (66.30%)	122 (76.30%)
Second	33 (20.60%)	22 (13.8%)
Third	11 (6.90%)	14 (8.80%)
Fourth or after	10 (6.25%)	2 (1.30%)
*Week of birth (M ± SD)*	31.48 ± 3.06	39.45 ± 1.70
*Infant in the NICU* ^*a,c*^		
Yes	135	N/A
No	10	N/A
Duration of NICU Stay in Weeks (N/%)^*a, b*^		
< 1 week	1(0.69%)	N/A
1 to 4 weeks	67(46.21%)	N/A
5 to 10 weeks	45 (31.03%)	N/A
11 to 15 weeks	16 (11.03%)	N/A
16 or more weeks	6(4.14%)	N/A

***N/B.***

^a^ This option was not presented to participants in the term group.

^b^ Participants (*n* = 15) from the second dataset were not presented with these questions. N/percentages are therefore representative of the *n* = 145 participants who were presented these questions.

### Configural invariance

The model showed a good fit for the data (CFI = 0.99, TLI = 0.98, RMSEA = 0.03 [90%CIs 0.000 to 0.048], SRMR = 0.07), showing the factor structure was consistent across groups.

### Metric invariance

The PSAS-RSF did not reach cut-offs for metric invariance (ΔRMSEA = 0.01, ΔCFI = 0.02, ΔSRMR = 0.01). Problematic items were identified by assessing differences in standardised regression coefficients between groups in the configural model (see Table 3).

**Table 3:** Standardised regression coefficients for the premature and term group

PSAS-RSF Factor	Item	Premature Group	Term Group	Difference
**Psychosocial Adjustment to Motherhood**	I have felt that I have had less control over my day than before my baby was born (PSAS-RSF 7)	0.67	0.52	0.15
	I have felt unable to juggle motherhood with other responsibilities (PSAS-RSF 2)	0.73	0.50	0.23
	I have worried that I am not going to get enough sleep (PSAS-RSF 12)	0.64	0.59	0.05
	I have worried more about my finances than before my baby was born (PSAS-RSF 10)	0.43	0.15	0.28
**Practical Infant Care Anxieties**	I have worried about my baby’s milk intake (PSAS-RSF 5)	0.57	0.51	0.06
	I have worried about my baby’s weight (PSAS-RSF 11)	0.56	0.48	0.08
	I have worried about the length of time my baby sleeps (PSAS-RSF 1)	0.58	0.60	-0.02
	I have worried about getting my baby into a routine (PSAS-RSF 14)	0.76	0.60	0.16
**Maternal Competence and Attachment Anxieties**	I have felt that my baby would be better cared for by someone else (PSAS-RSF 9)	0.56	0.40	0.16
	I have had negative thoughts about my relationship with my baby (PSAS-RSF 4)	0.72	0.49	0.23
	I have worried I will not know what to do when my baby cries (PSAS-RSF 16)	0.64	0.29	0.35
	I have worried that my baby is picking up on my anxieties (PSAS-RSF 3)	0.80	0.63	0.17
**Infant Safety and Welfare Anxieties**	I have repeatedly checked on my sleeping baby (PSAS-RSF 8)	0.63	0.65	-0.02
	I have worried that my baby will stop breathing whilst sleeping (PSAS-RSF 6)	0.71	0.70	-0.01
	I have felt frightened when my baby is not with me (PSAS-RSF 13)	0.71	0.23	0.48
	I have worried about my baby being accidentally harmed by someone or something else (PSAS-RSF 15)	0.72	0.40	0.32

Metric invariance was reached after the slope for one item was allowed to vary (*I have felt frightened when my baby is not with me;* ΔRMSEA = 0.01, ΔCFI=-0.01, ΔSRMR = 0.06*).* This model was also tested for scalar invariance, with loadings and intercepts for the problematic item being free to vary, and this was achieved (ΔRMSEA = 0.009, ΔCFI=-0.015, ΔSRMR = 0.006).

However, it is also notable that a comparison of the regression slopes between groups for three items with high differences in standardised regression coefficients were significant: *I have worried I will not know what to do when my baby cries* (*p* <.001); *I have worried about my baby being accidentally harmed by someone or something else* (*p* <.001); *I have worried more about my finances than before my baby was born* (*p* =.023), indicating these items may also be problematic.

## Discussion and conclusions

The aim of this study was to conduct a measurement invariance analysis to assess the performance of the PSAS-RSF in mothers of premature and term infants. Results demonstrate the PSAS-RSF achieves configural invariance, and so retains the original four-factor structure. However, it is clear there are several problematic items in the current version of the PSAS-RSF, which are being answered differently in mothers of premature infants.

The item* “I have felt frightened when my baby is not with me”* is being interpreted differently in mothers of premature infants on the PSAS-RSF. Extended periods of maternal-infant separation are relatively commonplace with premature infants, particularly at or shortly after the point of birth [33], and may contribute to feelings of anxiousness when separated. Admission to the neonatal intensive care unit [NICU] can contribute feelings of physical separation from the infant [34]. It may also be the environment of the NICU itself leads to feelings of separation, with parents often reporting the physical space around the infant is dominated by medical equipment, meaning they find it difficult to remain close to the infant [35]. Furthermore, the constraints of hospital policies mean mothers can feel further apart from their new baby [36]. This is important as prolonged separation, particularly considering the NICU, can lead to impairments in attachment and bonding [37, 38], which can increase anxiety [39].

Parental concerns for preterm infants also extend beyond their baby’s physical health. Financial concerns following the birth of a preterm baby are inversely associated with gestational age [40], and financial burdens of caring for a preterm baby are further exacerbated if the infant is in hospital for a prolonged period after birth, and can lead to a reliance on support networks [41]. Notwithstanding the financial burdens associated with hospital care immediately after birth, significant costs can persist right across the first year postpartum. For example, infants with severe health complications as a result of their birth may require heightened medical care once at home, including specialised equipment required to further support development, and follow-up appointments, which may lead to time off work extending beyond parental leave [42]. Furthermore, the NICU has been associated with significant out-of-pocket expenses, including childcare for other siblings, and direct medical costs which cause significant concern even in mothers who have paid parental leave [43]. It is also important to consider, as this study was international, the cost of healthcare may also be a contributing factor. For example, estimates suggest privately insured families in the United States may still incur out-of-pocket costs relating to NICU care in excess of $10,000 [44]; and in the UK, costs can reach approximately £75,000 for the earliest preterm infants, with all of this cost absorbed to the National Health Service [NHS] [45].

Anxieties surrounding infant health and accidental harm, as well as concerns surrounding infant care may also be increased in women with babies in NICU. They have already been found to be more prevalent for mothers with the earliest preterm infants [7]. The environment and constraints of the NICU, including handing over routine care of the infant to medical professionals can lead mothers to feel powerless and disconnected from their infant [19], which can further contribute to anxiety, leading mothers to feel uncertain about their parental role. This can lead to feelings of over-protection towards the infant [45], including fears regarding accidental harm and health. Mothers often feel uncertain about their parental role [46], and so may feel unsure and alone when the environment of the NICU is removed [47].

### Limitations

This study is the first to conduct a measurement invariance analysis of the PSAS-RSF across mothers of term and premature infants. However, the sample were predominantly White, married women from the United Kingdom of Great Britain and Northern Ireland and the Republic of Ireland. Future research should make a concerted effort to recruit women from diverse backgrounds, particularly as anxiety levels amongst mothers of premature infants can differ by race [48], and women from minority ethnic groups are less likely to engage with perinatal mental health services [49]. The results of the current study can inform modification of the current version of the PSAS-RSF for use in mothers of premature infants. Adaptation of the scale can be further informed by stakeholders and mothers of premature infants, before ensuring validity and reliability of any new measure.

### Conclusion

We conducted a measurement invariance analysis of the PSAS-RSF across mothers of term and premature infants. Results support the retention of the four-factor structure found in other variations of the PSAS as the measure achieved configural invariance, however, potentially problematic items include those around infant care, safety, and psychosocial adjustment to motherhood. Future research should now modify the PSAS-RSF for use in this specific population of mothers of premature infants.

## Data Availability

The dataset analysed for this study is not publicly available. However, reasonable requests for data can be sent to the corresponding author.

## Abbreviations

WHO: World Health Organization
PSAS: Postpartum Specific Anxiety Scale
PSAS-RSF: Postpartum Specific Anxiety Scale Research Short Form
PSAS-RSF-C: Postpartum Specific Anxiety Scale Research Short Form for Global Crisis
NICU: Neonatal Intensive Care Unit
TLI: Tucker Lewis Index
CFI: Comparative Fit Index
RMSEA: Root Mean Square Error of Approximation
SRMR: Standardised Root Mean Square Error of Approximation
